# Stabilization of the Central Part of Tropomyosin Molecule Alters the Ca2+-sensitivity of Actin-Myosin Interaction

**Published:** 2013

**Authors:** D.V. Shchepkin, A. M. Matyushenko, G. V. Kopylova, N. V. Artemova, S. Y. Bershitsky, A. K. Tsaturyan, D. I. Levitsky

**Affiliations:** Institute of Immunology and Physiology, Russian Academy of Sciences, Pervomayskaya Str., 106, Yekaterinburg, Russia, 620049; Bach Institute of Biochemistry, Russian Academy of Sciences, Leninsky prosp., 33, Moscow, Russia, 119071; Institute of Mechanics, Lomonosov Moscow State University, Michurinsky prosp., 1, Moscow, Russia, 119992; Belozersky Institute of Physico-Chemical Biology, Lomonosov Moscow State University, Leninskie gory, 1, bld. 40, Moscow, Russia, 119991

**Keywords:** regulation of muscle contraction, actin-myosin interaction, in vitro motility assay, tropomyosin

## Abstract

We show that the mutations D137L and G126R, which stabilize the central part of the tropomyosin (Tm) molecule, increase both the maximal sliding velocity of the regulated actin filaments in the *in vitro *motility assay at high Са^2+^ concentrations and the Са^2+^-sensitivity of the actin-myosin interaction underlying this sliding. Based on an analysis of the recently published data on the structure of the actin–Tm–myosin complex, we suppose that the physiological effects of these mutations in Tm can be accounted for by their influence on the interactions between the central part of Tm and certain sites of the myosin head.

## INTRODUCTION

Tropomyosin (Tm) is one of the key components of the regulatory apparatus of thin filaments in all types of muscles. According to the ‘steric blocking’ theory underlying the advanced concept of the regulatory mechanism of contraction of skeletal and cardiac muscles, Tm is capable of opening or closing the sites of actin interaction with myosin heads by shifting over the surface of an actin filament [1]. The Tm molecule is a dimer of α-helices forming a left-handed superhelix (‘coiled-coil’) [2]. Evidence has recently been obtained showing that the structure of the Tm molecule is not as simple as it has been considered so far. Extraordinary features specific only to Tm, such as the presence of sites with increased conformational mobility (flexibility), were observed. The conserved non-canonical residues Asp137 [3] and Gly126 [4], which disrupt the coiled-coil structure, were found in the central part of the Tm molecule. Replacement of these residues by canonical ones (mutations D137L, G126R and G126A) resulted in the stabilization of this part of the Tm molecule and completely prevented trypsin cleavage of Tm at the nearby Arg133 [3, 4]. Moreover, it was shown in both papers that the stabilizing mutations D137L and G126R (but not G126A) at high calcium concentrations (*p*Ca ≤ 5) cause a significant increase in the actin-activated ATPase activity of myosin heads during their interaction with actin filaments containing Tm and troponin, although having no effect both on the Ca^2+^-sensitivity of the myosin ATPase and on the Tm affinity for actin [3, 4]. In the present work, a thorough study of the effects of these mutations on the Tm regulatory properties was conducted. An *in vitro *motility assay, a highly sensitive method allowing one to monitor the sliding velocity of the reconstituted thin filaments over the surface covered with immobilized myosin, was used for this purpose for the first time.

## EXPERIMENTAL

Recombinant skeletal muscle α-Тms with the mutations D137L and G126R were prepared as described previously [4] using Tm with the mutation C190A in which Cys190 was replaced by Ala as a ‘wild type’ protein [3]. The experiments and the measuring of the sliding velocities of the regulated thin filaments with the* in vitro *motility assay at different Ca^2+^ concentrations were performed according to the described method [5]. A flow cell coated on the inside with nitrocellulose was filled with a solution of rabbit skeletal muscle myosin at a concentration of 0.5 μM (0.2 mg/ml); unattached myosin was subsequently washed out, and the regulated thin filaments were added into the cell. The filaments consisted of 10 nM F-actin labeled with rhodamine phalloidin, 0.1 μ M troponin, and 0.1 μ M Tm in a buffer containing 25 mM KCl, 25 mM imidazole, 2 mM ATP, 4 mM MgCl_2_, 1 mM EGTA, 20 mM DTT , 3.5 mg/ml glucose, 20 μ g/ml catalase, and 0.15 mg/ml glucose oxidase, pH 7.5 (these conditions are optimal for studying the sliding of the reconstituted thin filaments in an *in vitro *motility assay [6]). Free calcium concentrations were set by EGTA/CaEGTA in proportions calculated with the WebMaxC Standard program (http://www.stanford.edu/~cpatton/webmaxc/webmaxcS.htm). The experiments were conducted at 30°C; the sliding velocity of the filaments was measured using the GMimPro software [7].

## RESULTS AND DISCUSSION

**Fig. 1:**
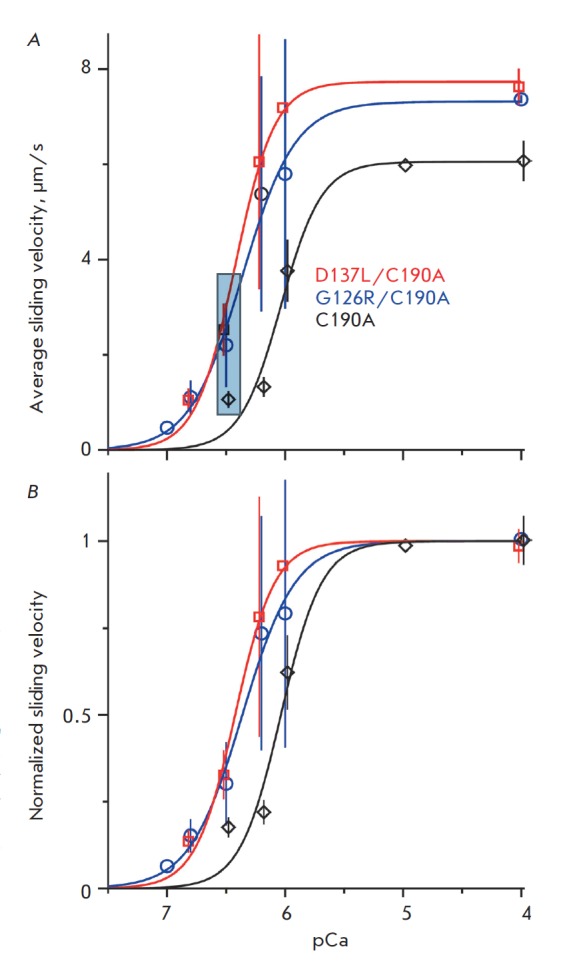
The average sliding velocity of the thin filaments along the myosin-coated surface as a function of the Ca^2+^ concentration. *A*: average data for 2–3 experiments with each of the Tm mutants. Vertical lines show the standard deviations. *B*: the same data as in *A *normalized for the maximum velocity

The results of the experiments indicate that the D137L and G126R mutations, which stabilize the central part of Tm, not only enhance the maximum sliding velocity of the regulated thin filaments in the *in vitro *motility assay at high Ca^2+^ concentrations (Fig. 1A), but also increase the Ca^2+^-sensitivity of the velocity by shifting the calcium-velocity curve towards lower Ca^2+^ concentrations (Fig. 1B). The pCa50 value (i.e. the negative logarithm of the concentration of free Ca^2+^ at which the sliding velocity is half-maximal) was 6.06 ± 0.04 (here and onwards mean ± SEM) for the regulated thin filaments containing the ‘reference’ Tm with the C190A mutation. The value was equal to 6.36 ± 0.05 for the filaments containing Tm with the mutations D137L/C190A and 6.42 ± 0.03 for the filaments with the Tm mutant G126R/C190A. Thus, we have demonstrated for the first time that the mutations D137L and G126R stabilizing the central part of the Tm molecule significantly increase the Ca^2+^-sensitivity of the actin-myosin interaction underlying the molecular mechanism of muscle contraction, which is regulated by changes in the Ca2+ concentration within muscle fiber. The data on the increase in the filament sliding velocity in the *in vitro* motility assay (Fig. 1A) correlate well with the increase in the myosin ATPase rate in the presence of regulated thin filaments with the mutations D137L and G126R in the central part of Tm at a saturating Ca^2+^ concentration [3, 4].

**Fig. 2:**
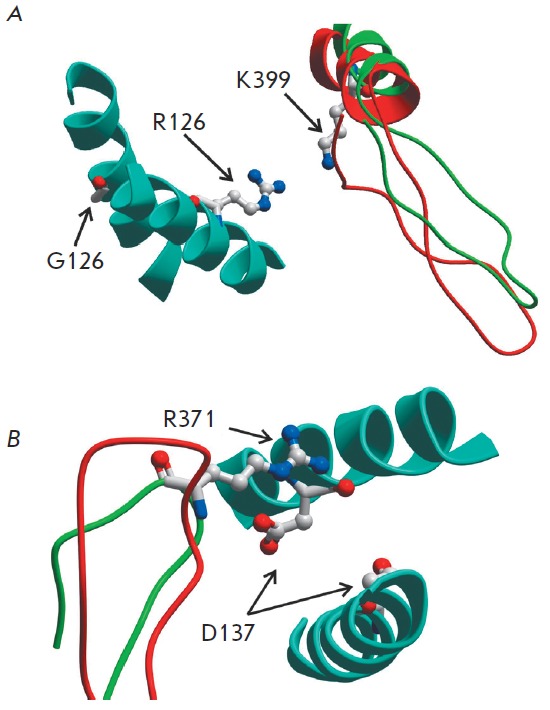
The structural model of the contact area of a myosin head strongly bound to actin with Tm on the surface of the thin filament. Only some parts of the myosin head adjacent to the amino acid residues 126 (*A*) and 137 (*B*) in the central part of Tm are shown. Actin and other parts of myosin and Tm are not shown. The model was obtained from the structure of the actin–myosin–Tm complex ([8], pdb code 4A7H) by superimposing the upper 50-kDa domain of the myosin head of chicken fast skeletal muscle myosin II (pdb code 2MYS) instead of the same domain of myosin-I used in [8]. Segments of the Tm double α-helix are shown by blue ribbons, the parts of the myosin-I head used in [8] are shown in red, and those of the head of skeletal muscle myosin-II are green. The residue R126 of the Tm G126R mutant (*A*) and a ‘non-canonical’ Tm residue D137 (*B*), which was replaced with Leu in the Tm mutant D137L/C190A, as well as the charged myosin residues K399 (*A*) and R371 (*B*) located in close proximity to these Tm residues are shown in a ‘ball-and-stick’ atomic representation. The distance between the charged atoms of myosin residue K399 and Tm residue R126 (*A*) in the model was 8.8 A^°^, and that between myosin R371 and Tm D137 (*B*) was 4.7 A^°^. The model and the picture were prepared using ICM-Browser (MolSoft, CA, USA)

In order to interpret the results, we chose a new approach based on an analysis of recent data regarding the structure of the actin–Tm–myosin complex obtained using cryo-electron microscopy with a 8 A^°^ resolution [8]. An important feature of this structure is the presence of direct contacts between Tm located on the surface of the actin filament and some areas of the myosin heads. Since this structure was obtained with a non-muscle myosin-I, our model (Fig. 2) was built by replacing the Tm-interacting domain in myosin-I with the corresponding domain of the skeletal muscle myosin-II used in our experiments. In this model, we searched for the residues in the myosin head that are sufficiently close to the residues at the positions 126 and 137 in the central part of Tm in order to be able to interact with them. The results of the search are shown in Fig. 2. It turned out that a small Gly126 is incapable of any interaction with myosin. However, due to the G126R mutation, the side chain of Arg126 in Tm is in the vicinity of that of residue Lys399 in the myosin head, so that an electrostatic repulsion emerges between the positively charged atoms of these residues (Fig. 2A). Such interpretation is supported by the fact [3] that the replacement of Gly126 in Tm with a small hydrophobic Ala, not charged Arg, did not affect the ATPase activity of myosin in the presence of regulated actin filaments. On the other hand, the negative charge of Asp137 is close to the positive charge of Arg371 of the myosin head and electrostatically interacts with it (Fig. 2B). This interaction is violated by replacing the charged residue Asp137 with a neutral Leu residue in the D137L mutant.

Thus, the investigated mutations in the central part of the Tm molecule in both cases should lead to a decrease in the energy of interaction between Tm and the myosin head strongly bound to actin. The magnitude of the energy reduction is small compared to the energy of the strong myosin binding to actin [8] but is comparable to the energy required to move Tm over the surface of the actin filament. According to the steric blocking theory [1, 9], in the absence of Ca^2+^ troponin keeps Tm on the actin filament at a position in which it covers the myosin binding sites on actin. When the Ca^2+^ concentration increases, troponin detaches from actin and Tm moves aside, slightly opening the myosin binding areas. The myosin heads first attach to actin ‘weakly’ and not tightly, and then go into the ‘strongly’-bound state and shift the Tm chain further away, thus opening the neighbor myosin binding sites on the adjacent actin monomers. According to our data, the explored mutations which stabilize the central part of Tm facilitate its displacement over the surface of the actin filament by strongly bound myosin heads and accordingly allow a greater number of neighboring myosin heads to bind actin and to produce mechanical work. This can probably explain the noticeable effect of such mutations in the Tm molecule on the sliding velocity of regulated thin filaments and the Ca^2+^-sensitivity of their sliding in the *in vitro *motility assay.

## Abbreviations

Tm: tropomyosin

## References

[1] McKillop D.F.A., Geeves M.A. (1993). Biophys. J..

[2] Nevzorov I.A., Levitsky D.I. (2077). Biochemistry (Moscow)..

[3] Sumida J.P., Wu E., Lehrer S.S. (2008). J. Biol. Chem..

[4] Nevzorov I.A., Nikolaeva O.P., Kainov Y.A., Redwood C.S., Levitsky D.I. (2011). J. Biol. Chem..

[5] Shchepkin D.V., Kopylova G.V., Nikitina L.V., Katsnelson L.B., Bershitsky S.Y. (2010). Biochem. Biophys. Res. Commun..

[6] Homsher E., Kim B., Bobkova A., Tobacman L.S. (1996). Biophys. J..

[7] Mashanov G.I., Molloy J.E. (2007). Biophys. J..

[8] Behrmann E., Müller M., Penczek P.A., Mannherz H.G., Manstein D.J., Raunser S. (2010). Cell..

[9] Lehman W., Craig R. (2008). Adv. Exp. Med. Biol..

